# *Kcnq*1-5 (Kv7.1-5) potassium channel expression in the adult zebrafish

**DOI:** 10.1186/1472-6793-14-1

**Published:** 2014-02-20

**Authors:** Calvin Wu, Kanishk Sharma, Kyle Laster, Mohamed Hersi, Christina Torres, Thomas J Lukas, Ernest J Moore

**Affiliations:** 1 Department of Molecular Pharmacology & Biological Chemistry, Northwestern University, Chicago, IL 60611, USA; 2 Department of Biological Sciences, University of North Texas, Denton, TX 76203, USA; 3 Department of Speech & Hearing Sciences, University of North Texas, Denton, TX 76203, USA

**Keywords:** Zebrafish (Danio rerio), *kcnq1-5*, RNA transcripts, Kcnq protein, Zebrafish genome, qRTPCR, Tinnitus

## Abstract

**Background:**

*KCNQx* genes encode slowly activating-inactivating K^+^ channels, are linked to physiological signal transduction pathways, and mutations in them underlie diseases such as long QT syndrome (*KCNQ*1), epilepsy in adults (*KCNQ*2/3), benign familial neonatal convulsions in children (*KCNQ*3), and hearing loss or tinnitus in humans (*KCNQ*4, but not *KCNQ*5). Identification of *kcnqx* potassium channel transcripts in zebrafish (Danio rerio) remains to be fully characterized although some genes have been mapped to the genome. Using zebrafish genome resources as the source of putative *kcnq* sequences, we investigated the expression of *kcnq1-5* in heart, brain and ear tissues.

**Results:**

Overall expression of the *kcnq*x channel transcripts is similar to that found in mammals. We found that *kcnq1* expression was highest in the heart, and also present in the ear and brain. *kcnq2* was lowest in the heart, while *kcnq3* was highly expressed in the brain, heart and ear. *kcnq5* expression was highest in the ear. We analyzed zebrafish genomic clones containing putative *kcnq4* sequences to identify transcripts and protein for this highly conserved member of the Kcnq channel family. The zebrafish appears to have two *kcnq4* genes that produce distinct mRNA species in brain, ear, and heart tissues.

**Conclusions:**

We conclude that the zebrafish is an attractive model for the study of the KCNQ (Kv7) superfamily of genes, and are important to processes involved in neuronal excitability, cardiac anomalies, epileptic seizures, and hearing loss or tinnitus.

## Background

Potassium channels are well-established biological targets for diseases including neuropathic pain, epilepsy, cardiac arrhythmia, hearing loss, deafness, or tinnitus [1]. In particular, mutations in the *KCNQ4* potassium gene and perhaps *KCNQ3* are associated with progressive high frequency hearing loss [2,3]. Of the several ion channels used by the sensory hair cell, the K^+^ channel KCNQ4 is thought to modulate the membrane potential of hair cells to adjust the sensitivity of hearing in a variety of mammals [1,4,5]. Similarly, KCNQ4 and KCNQ5 are key modulators of L-type Ca^2+^ channel activity in cardiovascular cells [6]. Variants of *KCNQ5* are not associated with sensory hearing loss in humans, but there is high abundance in the larval zebrafish ear [7,8], and thus, may be related to yet to be defined developmental factors related to hearing [9].

A recent study characterized the expression of *kcnq2*, *kcnq3*, and *kcnq5* in whole larval zebrafish (*Danio rerio)*[7], but we know little about the expression of the complement of *kcnq* genes and the K^+^ ion channels that they encode in various organs of the adult zebrafish. Since certain drugs and metal ions affect the function of Kcnq channels [10-12] in a dose-dependent manner, these agents can be used to alter ion permeability across the membrane of zebrafish hair cells and thus create a fish model of sensory cell dysfunction. KCNQ2-5 channels are also regulated by intracellular signal transduction effectors such as phospholipids [13], phosphorylation [14], and calmodulin [15]. However, little is known about how these signaling systems impact the *kcnq* channels in zebrafish sensory pathways. Thus, the zebrafish offers a unique opportunity to study Kcnq channel modulation, function and dysfunction.

The zebrafish has served as an especially attractive model for the study of the development and function of the vertebrate inner ear [8,16]. It has three methods of sensing sound within its environment. The first involves the lateral line system, which is comprised of a set of neuromasts containing hair cells arrayed along each side of the body. Neuromasts contain bundles of sensory hair cells beneath a cupula, which are responsible for sensing the displacement of water molecules [17]. The second means of sensing sound are structures of the inner ear composed of the utricle, saccule, lagena and pars neglecta. Each of these anatomical structures house patches of sensory hair cells and supporting cells that are embedded in the epithelial lining of the macula [18]. The hair cells found in these structures are similar to those found in mammals, and contain voltage gated and ligand gated ion channels presumably linked to several signal transduction pathways. Third, there are sets of motion detectors or neuromasts arrayed around the head, particularly the orbital regions. In this report, we have studied Kcnq channel expression and localization in several tissues of the zebrafish. Using the deduced mRNA sequences in the available databases, we probed for the presence of Kcnq channel mRNA transcripts in the ear, brain and heart, and partially characterized the amino acid sequence of one channel protein. The zebrafish genome has two different *kcnq4* genes, one of which has been localized to chromosome 19. The mRNA from this gene is also expressed in zebrafish brain and ear. We prepared a specific antibody to zebrafish Kcnq4, quantified its levels using qRT-PCR, and further verified its expression using Western blots of brain and ear tissues.

## Results

### Detection of Kcnq Expression in Zebrafish

Amplicons representing *kcnq1-5* were detected by RT-PCR analyses and observed under UV illumination. Table 1 shows the primers, amplicon size, and primer sequences used for all PCR reactions. Primers and nested primers were designed to cross several exons of the specific PCR template sequence.

**Table 1:** **Summary of primers designed to amplify *kcnq *1-5 and** β-**actin mRNAs**

**Primer**	**Nucleotide sequence**	**Expected size (bp)**
KCNQ1 fwd	5′- TCC AGT CGC TCA TGT GTC TC -3′	203
KCNQ1 rev	5′- TTT CAT CCC ACC TTC TTT GC -3′	
KCNQ2 fwd	5′- GAG CCA GTG CAG GAG AAA AG -3′	344
KCNQ2 rev	5′- TGA GGT AGA AGG CCG ACA CT -3′	
KCNQ3 fwd	5′- GAG AAG GAT TCG GCT CAC TG -3′	443
KCNQ3 rev	5′- GCG TCT GCA TAG GTG TCA AA -3′	
KCNQ4 (a) fwd	5′-TAT GCA GAC TCC CTC TGG TG-3′	175
KCNQ4 (a) rev	5′-CCT GCA CTT TCA GAG CAA AG-3′	
KCNQ4 (b) fwd	5′-GGG CCG CAG GGT TTC TTT AAA CTT-3′	400
KCNQ4 (b) rev	5′-ATG ACA GTA TGC TGC CGT CCT TCA-3′	
KCNQ4 (c) fwd	5′-CGG CCG CAG GGT TTC TTT AAA CTT-3′	400
KCNQ4 (c) rev	5′-TCC TTC AGT GGG AAG ATG GGC TTT-3′	
KCNQ4 ch19(a) fwd	5′-TGC CTG TAC AAT GTG CTG GAG AGA-3′	265
KCNQ4 ch19(a) rev	5′-AAG GCT TTC TGG CAA AGC GTA GTC-3′	
KCNQ4 ch19(b) fwd	5′-ATC AGC CAA TGA TGA CAG ACG GGT-3′	458
KCNQ4 ch19(b) rev	5′-AAG GCT TTC TGG CAA AGC GTA GTC-3′	
KCNQ5b fwd	5′- TGC CTG GTA TAT TGG GTT CC -3′	261
KCNQ5b rev	5′- TGA ACC TTC AAG GCA AAA CC -3′	
β-actin fwd	5′- TCC CCT TGT TCA CAA TAA CC- 3′	350
β-actin rev	5′- TCT GTG GCT TTG GGA TTC A-3′	

**Figure 1:**
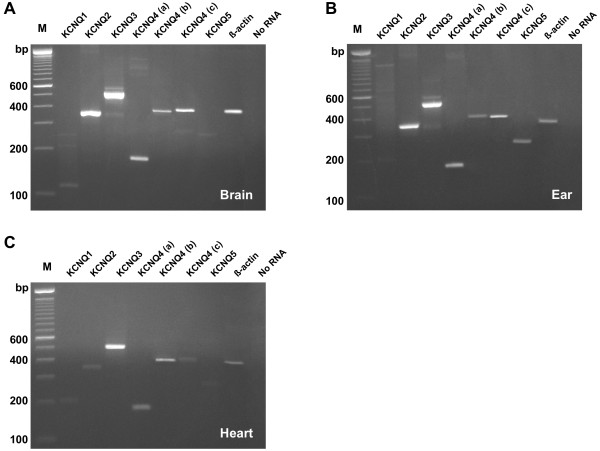
**Expression of *kcnq *in zebrafish brain (A), ear (B) and heart (C).** Lanes labeled M are the 100 bp ladder molecular standards. Lane 9 and 10, respectively, is a positive control with β-actin, and a negative control without RNA.

**Figure 2:**
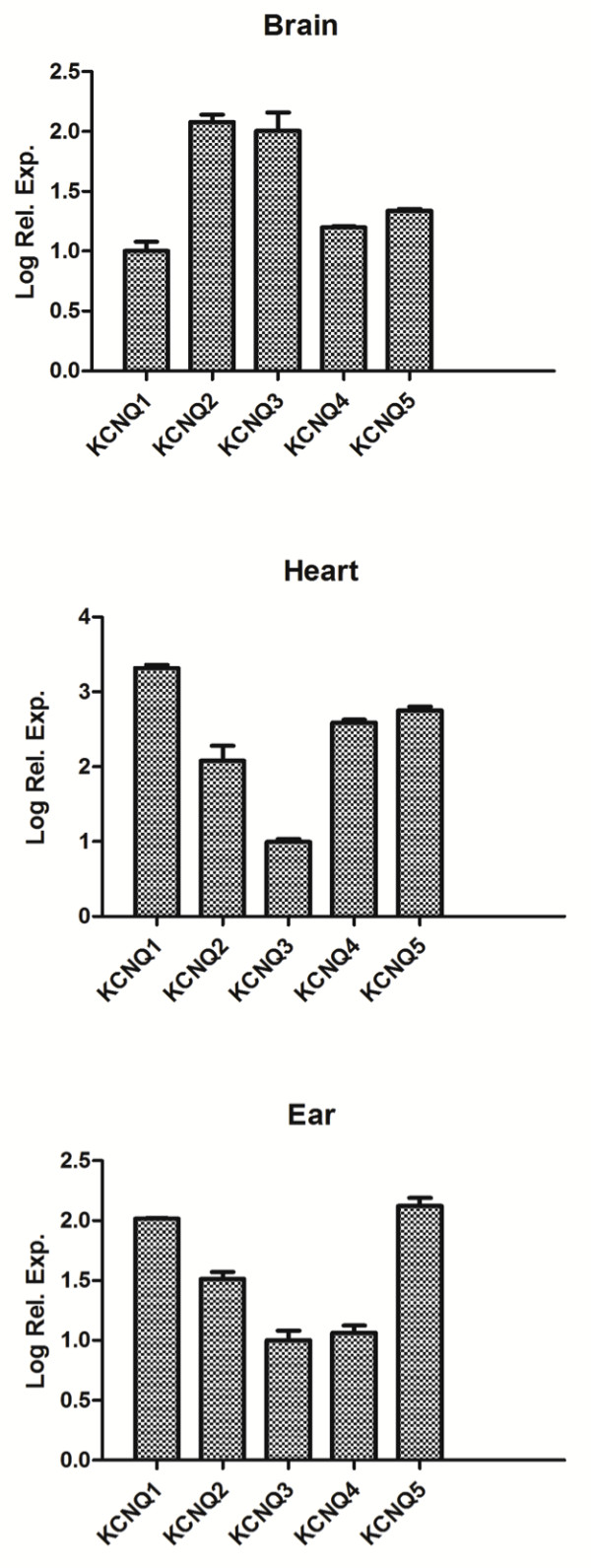
**qRTPCR of Kcnq channel transcripts in brain, heart, and ear.** Real time PCR thresholds (Delta CT) were first normalized to GAPDH or β-actin for each tissue. These data were then plotted relative to the lowest expressed mRNA in each tissue using a logarithmic scale. Depicted also is +/-1.0 standard error.

Several of the *kcnq* RNA transcripts were expressed in the zebrafish brain (Figure 1A) consistent with observations in other species [19]. *Kcnq1* and *kcnq*5 were absent in the gel (Figure 1A), but were detected in the quantitative data (Figure 2, top), at the same approximate levels. The PCR data for *kcnq5* were done with *kcnq5b* primers while the quantitative PCR was done with *kcnq5a* primers. *Kcnq*4 was probed by three different sets of primers (KCNQ4-a, KCNQ4-b, KCNQ4-c) for downstream cDNA sequencing. Bands for *kcnq4* for the three sets of primers were readily detected. *Kcnq2* and *kcnq3* showed the strongest signal, while *kcnq*3 displayed two almost overlapping bands. A negative control (PCR reaction without RNA, or “No RNA”) is shown in the last lane. The expression strength of the mRNA transcript was compared to the intensity of the *β*-actin control.

The inner ear tissue of zebrafish included the sensory epithelium (culled from 6 fish, both ears), consisting also portions of the utricle, saccule and lagena tissues, but not semicircular canals. Figure 1B shows mRNA expression of *kcnq1-5* in the zebrafish ear - all *kcnq* transcripts were detected. However, *kcnq1* was somewhat weak, while *kcnq5b* provided a much stronger signal. Figure 1C shows the expression pattern in zebrafish heart. Except for *kcnq5b*, transcripts for *kcnq1-4* were detected.

**Figure 3:**
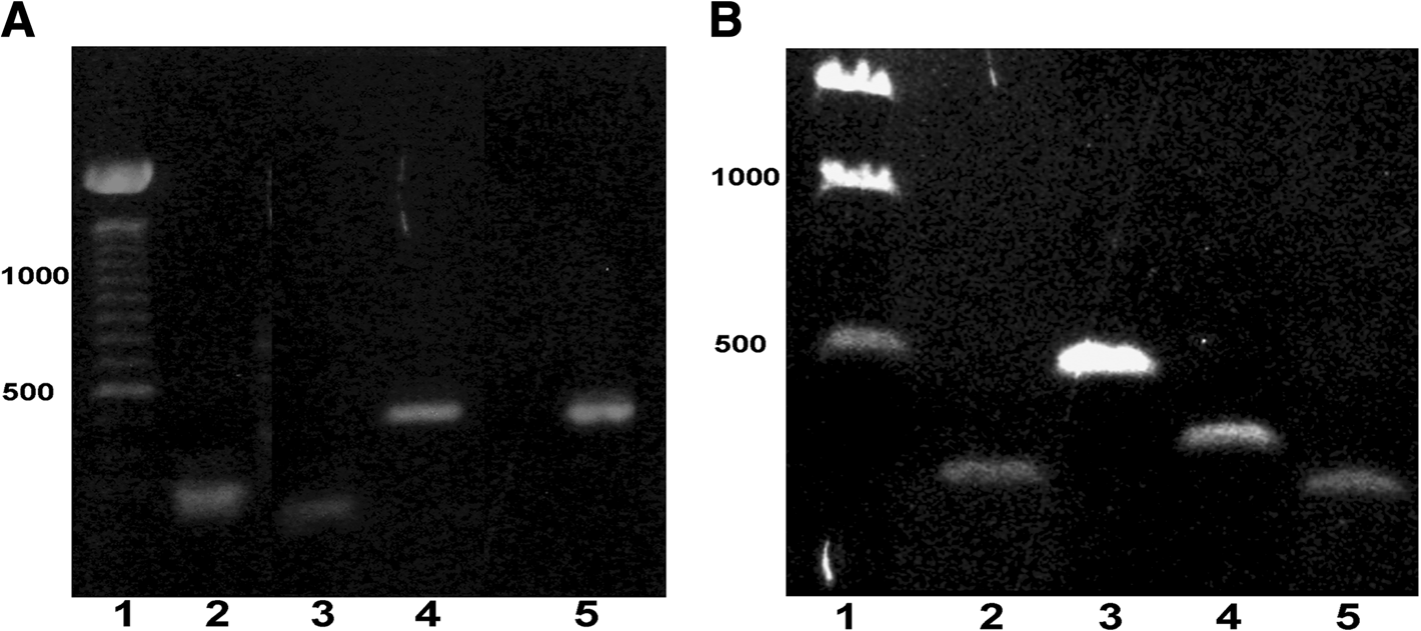
**Expression of *kcnq4 *transcripts from chromosome 19. A**. PCR products from reverse transcriptase PCR reactions using primers (Table 1) for *kcnq*4. Lanes 2 and 5 are brain mRNA products, while lanes 3 and 4 are zebrafish ear mRNA-derived products. Lane 1 is a 100 bp ladder standard. **B**. PCR products from reverse-transcriptase PCR reactions using primers (Table 1) for Chr 19 (Lanes 2 and 3) and the Zv9_NA546 scaffold *kcnq*4 (lanes 4 and 5) of mRNA from zebrafish heart. Lane 1 is a 500 bp ladder standard.

**Figure 4:**
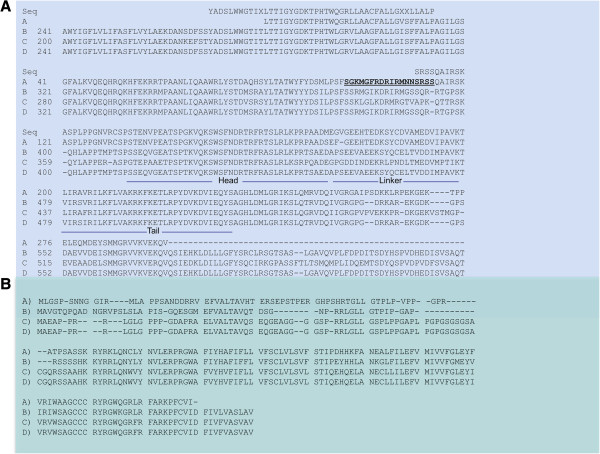
**A. Alignment of the partial zebrafish *kcqn4 *sequence (A), with KCNQ4 sequences from chimpanzee (B), frog (C) and human (D).** The sequences on top of the KCNQ4 groups are the translation of the sequences that we obtained from the sequencing of PCR products (Table 2). The carboxyl region that contains the sequences implicated in the assembly of homo- vs. hetero-tetramers is indicated with bars, with the Head-Linker-Tail designations. **B**. Alignment of the translation of the Chr 19 sequence of zebrafish Kcnq4 (A) with the amino termini of Frog (B), Chimpanzee (C) and Human (D).

As mentioned in the Introduction, the partial sequence of a *kcnq4* gene has also been mapped to chromosome 19. We detected expression of transcripts based upon this gene in brain and ear (Figure 3A) and the heart (Figure 3B). The sequence is located at the 5′ end (519 bp) of the transcript encoding a 173 amino acid sequence, homologous to *KCNQ4* from human as well as other species (See Figure 4). Using various combinations of primers in the RT-PCR experiments, all attempts to link sequences of the chromosome 19 transcripts to the more 3′ *kcnq4* sequences in our mRNA, which is not yet mapped to a chromosome, failed to show up in our data (not shown). Therefore, we conclude from these observations that there are two separate *kcnq4* genes expressed in the zebrafish.

### qRTPCR of kcnq1-5 expression in brain, ear, or heart

End point PCR is not applicable to quantitative measures of expression, and detection of bands can be variable using electrophoretic separation. Therefore, we performed qRTPCR using reverse-transcribed mRNA templates from each tissue. Different primers were designed to produce amplicons (100-200 bp) suitable for SYBR green-based real time quantitative analysis (Table 2). As shown in Figure 2, the brain has very high expression of *kcnq2* and *kcnq3* compared to other tissues. *Kcnq2-5* transcripts are lower than *kcnq1* in the heart, while *kcnq5*a is particularly elevated in the ear, when compared to the brain.

**Table 2:** Partial sequences of KCNQ4 obtained from PCR products

**KCNQ4 (a) primers**					
5′-					
NNNNNNNNNN	CGGGCAGGGC	AAGAAGNNCT	CCCAGAAGGG	CAAAACAAGC	TGCTAAAAGA
CGACCTTGCC	AGGTGTGTGG	AGTCTTGTCA	CCGTAGCCGA	TCGTAGTCAG	GGTTATCGTC
CCCCACCAGA	GGGAGTCTGC	ATAA			
-3′ (144 bp)					
**KCNQ4 (b) primers**					
5′-					
NNNNNNTNNG	ATGATATTCT	CGCTCCTCTC	AGGCCATCCG	GAGCAAGGCT	TCTCCTTTA
CTCCAGGTAA	CGTGCGGTGT	TCACCCAGCA	CTGAGAACGT	CCCAGAAGCC	ACCAGCCCTG
GGAAAGTGCA	GAAAAGCTGG	AGCTTCAATG	ACCGGACACG	TTTTCGCACA	TCTCTGCGCC
TCAAACCACG	ACCCGCTGCA	GACATGGAGG	GAGTCGGAGA	AGAGCACACT	GAGGACAAAT
CTTACTGTGA	CGTGGCCATG	GAGGATGTGA	TTCCCGCAGT	GAAGACCCTG	ATTCGAGCGG
TTCGGATCCT	GAAGTTCCTG	GTGGCCAAGA	GGAAGTTTAA	AGANCCCCTT	GCCGGCCGAA
NNG					
-3′(363 bp)					
**KCNQ4 (c) primers**					
5′-					
CNNNNNNTNG	GATGATNTTC	TCGCTCCTCT	CAGGCCATCC	GGAGCAAGGC	TTCTCCTTTA
CCTCCAGGTA	ACGTGCGGTG	TTCACCCAGC	ACTGAGAACG	TCCCAGAAGC	CACCAGCCCT
GGGAAAGTGC	AGAAAAGCTG	GAGCTTCAAT	GACCGGACAC	GTTTTCGCAC	ATCTCTGCGC
CTCAAACCAC	GACCCGCTGC	AGACATGGAG	GGAGTCGGAG	AAGAGCACAC	TGAGGACAAA
TCTTACTGTG	ACGTGGCCAT	GGAGGATGTG	ATTCCCGCAG	TGAAGACCCT	GATTCGAGCG
GTTCGGATCC	TGAAGTTCCT	GGTGGCCAAG	AGGAAGTTTA	AAGAAACCCT	GCGGCCGA
-3′ (358 bp)					

### Kcnq4 *protein expression using Western Blots*

**Figure 5:**
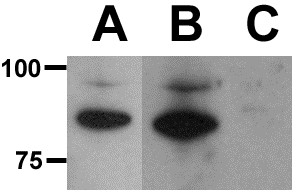
**Kcnq4 protein expression in zebrafish brain and ear tissues.** Western blots of Brain (Lane A) and Ear (Lane B) extracts probed with rabbit antibody to zebrafish Kcnq4. Lane C is zebrafish ear tissue probed with antisera containing 100 ng/mL of the synthetic peptide used for immunogen production added.

To further verify the presence of Kcnq4 protein, we probed Western blots of ear (Figure 5, lane A) and brain (Figure 5, lane B) tissue with rabbit polyclonal antipeptide antisera. A major band at ~80 kDa corresponding to the size of Kcnq4 in mammalian species was readily detected in ear, and brain tissue extracts. The band was absent (Figure 5, lane C) when the immunizing peptide was present along with the primary antibody. Pre-immune sera from the rabbit showed no reactivity to proteins in zebrafish brain or ear tissue extracts (not shown). Using appropriately designed primers, we also performed DNA sequencing on the *kcnq4* PCR products that are summarized in Table 2. The sequenced data maps to various zebrafish genomic clones, and the translation of the mRNA provide amino acid sequences consistent with KCNQ4 (Figure 4), indicating that the clones represent active transcripts of a Kcnq4 protein.

### *Genetic and comparative analysis of* Kcnq4 *Proteins (channels)*

**Figure 6:**
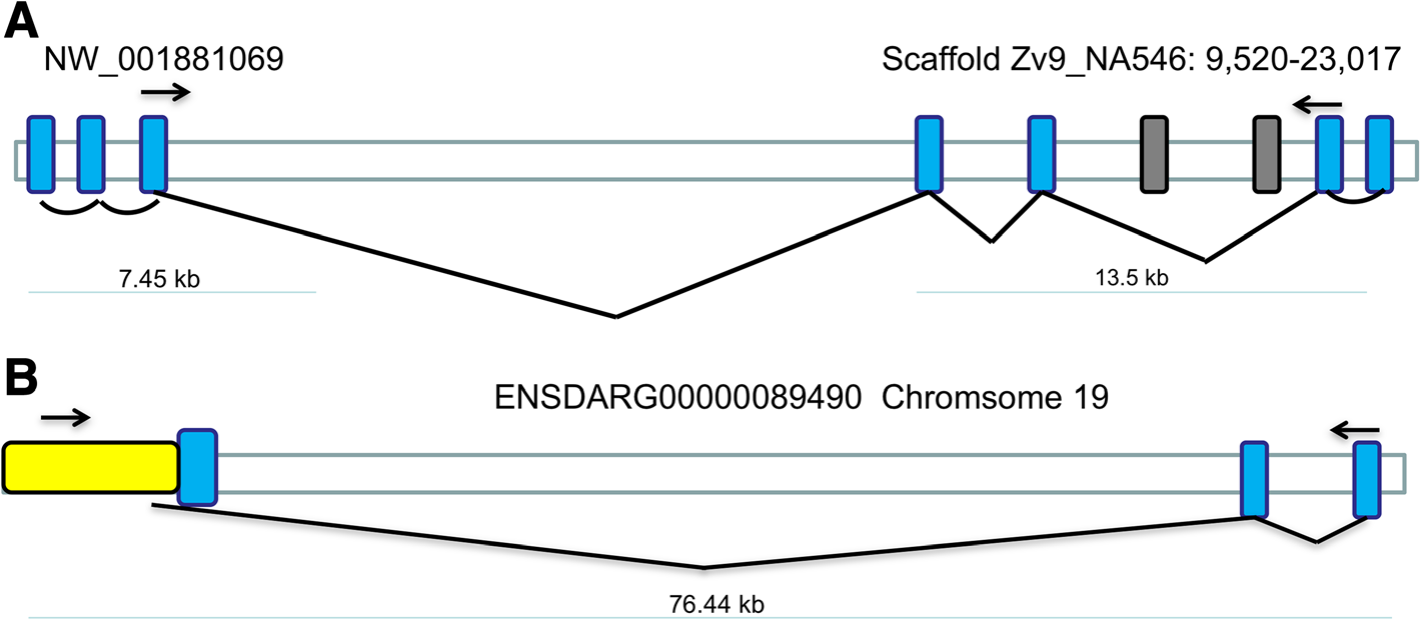
**Analysis of *kcnq4 *genomic structures. A**. Schematic representation of zebrafish genomic clone containing *kcnq4*-related sequences - these have not at the time of the publication of this work been mapped to a chromosome. **B**. Partial structure of the Chr 19 Kcnq4 genomic clone. Only the first three exons have been identified. Blue boxes indicate active exons, while white boxes indicate silent exons. A yellow box shows the 5′ untranslated region of Kcnq4 from chromosome 19. Arrows indicate the location of primers used for mRNA detection across the largest number of exons.

We checked the putative *kcnq4* cDNA sequence against the zebrafish genome assembly and clones by BLAST searching. We retrieved the sequences of two genomic clones that contained *kcnq4* sequences (Figure 6A). Using two exon prediction programs, Net2Gene [20] and HMM [21], we generated a mRNA sequence comparable to the GenBank sequence, except that the 300 bp of 5′ end and the 200 bp of 3′ end sequence were not found. In the genomic clones, NW_001881069 contains three exons, while the scaffold Zv9_NA546:9, 520-23,017 contains 4 exons. We originally analyzed an earlier sequence deposited in GenBank (NW_00188744) that contains the same exons as the scaffold Zv9_NA546: 9,520-23,017. Two cryptic exons in NW_00188074 detected by the exon predictions programs are not used in the zebrafish Kcnq4. Moreover, ORFs within these two cryptic exons do not contain amino acid sequences related to the Kcnq family of channels. For comparison, the exon structure of the partial *kcnq*4 gene from chromosome 19 is shown in Figure 6B. Our PCR data confirmed that the transcript contains a sequence from the three exons in this partial gene.

The derived coding sequence from the 7 exons of NW_001881069 + Zv9_NA546: 9, 520-23,017 matches very well to other Kcnq4 amino acid sequences from chimpanzee, frog, and humans (Figure 4A). Similarly, the translation of the 5′ mRNA from chromosome 19 KCNQ4 is also very well conserved in the same species (Figure 4B). Table 3 summarizes all of the known Kcnq genes in zebrafish as of January 2013. *Ensembl* IDs are used for the gene, mRNA, and translated protein sequences, as these are referenced directly in the ZFIN database. Cross-references to entries in GenBank and Uniprot are also tabulated. *Kcnq1* on chromosome 7 has three potential splice products of differing lengths that have not been fully characterized. *Kcnq1* also has another gene identified on chromosome 25, but no transcripts have yet to be reported. *Kcnq2* has two genes, one on chromosome 6 that we also detected in this work, and another on chromosome 8 that was detected in zebrafish larvae [7]. *Kcnq5* has also two genes, and in this paper, we focused mostly on transcripts derived from chromosome 13 (*kcnq5a*). The chromosome 1 derived *kcnq5b* has also been detected in zebrafish larvae [7].

**Table 3:** **Summary of *kcnq *** **genes in zebrafish (2013)**

**Name**	**Chr.**	**Gene**	**mRNA**	**Protein**	**RefSeq**	**UniProt**
*kcnq1*	25	ENSDARG 00000088397				
*kcnq1*	7	ENSDARG 00000059798	ENSDART 00000125483	ENSDARP 00000106445		A1L1U6
	7	ENSDARG 00000059798	ENSDART 00000083516	ENSDARP 00000077951		F1QG65
	7	ENSDARG 00000059798	ENSDART 00000083516	ENSDARP 00000077949	NP_001116714	B0R0K2
					NM_001123242	
*kcnq2a*	8	ENSDARG 00000075307	ENSDART 00000131736	ENSDARP 00000122368		B8JIR6
*kcnq2*	6	ENSDARG 00000091130	ENSDART 00000130440	ENSDARP 00000107870	XM_003198845	E7F4W4
					XP_00319889	
*kcnq3*	2	ENSDARG00000060085	ENSDART00000084303	ENSDARP 00000078738	XM_003197933	F1RE25
					XP_003197981	
*kcnq4*	Zv9 NA546	ENSDARG00000089559	ENSDART 00000125605	ENSDARP 00000108227		
*kcnq4′*	19	ENSDARG00000089490	ENSDART 00000129915	ENSDARP 00000108792		
*kcnq5a*	13	ENSDARG 00000069954	ENSDART 00000139904	ENSDARP 00000118905	XM_679763	B8JHS5, F1Q5A8
					XP_684855	
*kcnq5b*	1	ENSDARG 00000069953	ENSDART 0000085370	ENSDARP 00000079805	XP_697081	F1RB62
					XM_691989	

## Discussion

In this study, we characterized Kcnq-type proteins/channel expression in brain, heart, and ear tissues of the zebrafish. We show that members of the Kcnq (Kv7.x) family of mRNAs are present in these tissues. Further, we demonstrated mRNA as well as the protein for Kcnq4 in ear and brain extracts from adult zebrafish. Although signals for *kcnq1* and *kcnq5* were weak using end-point PCR, the transcripts were readily detected in all tissues using qRTPCR. These data are consistent with previous reports of the Kcnq1 channel expressed during development [22].

As previously found in mammals [1,3-5,23-25], *kcnq2* was expressed in zebrafish brain, heart and ear. Similarly, in mammals, *kcnq3* is usually found co-expressed in the same tissues. Kcnq4 was detected in ear and brain tissue using a Kcnq4 selective antibody. KCNQ4 is found in auditory hair cells in mammals and we suggest that it may be present in homologous cells in the zebrafish.

The amino acid sequences of zebrafish Kcnq4, as well as other members of the KCNQ channel family, are conserved across phylogeny [19]. One distinguishing characteristic of KCNQ2 and KCNQ3 is the presence of a clustering domain that allows interaction of KCNQ channels with Na^+^ channels in the nodes of Ranvier [26]. Another characteristic of KCNQ channels is that the structural assembly (homotetramer vs. heterotetramer) is dependent upon amino acid sequences in the carboxyl-terminal region [23]. In the case of zebrafish Kcnq4, the translated amino acid sequence that we derived (Figure 4A) is consistent with the head-linker-tail structure of KCNQ4 that supports a homotetrameric structure [23].

The more highly abundant KCNQ transcripts expressed in the brain (KCNQ2, KCNQ3, and KCNQ5) are possible contributors to a number of important electrophysiological functions that are necessary for normal cognitive function. That is, dysfunction of these channels has been associated with dementia, stroke, and epilepsy [24]. Very similar to the mammalian cochlea [4,25], but perhaps more similar to the vestibular system [27], our results show that the zebrafish inner ear sensory tissues do express the *kcnq*2-5 genes. The inhibition of KCNQ4 activity in the mammalian cochlea [28] or knockout mouse [29] causes sensory cell degeneration followed by deafness. However, unlike mammals, the zebrafish hair cells are capable of regeneration after acoustic or chemical insult [30,31], and selected transcription factors among other putative molecules are key mediators of the regeneration [32,33]. No variants of *KCNQ5* are associated with sensory hearing loss in humans so perhaps its high abundance in the zebrafish ear is associated with regenerative capabilities.

Studies of the effects of exogenous regulators of zebrafish hair cell regeneration are at various stages of investigation [34]. Our identification of Kcnq channels in zebrafish may offer a new *in vivo* model system for screening KCNQ channel modulators/drugs and their effects on regeneration. Certain classes of drugs are being designed to modulate the activity of specific KCNQ-type channels [35-37], and our work suggests that screening this class of chemotherapeutic agents for functional [38-40], as well as for adverse effects (such as behavioral abnormalities) in the zebrafish is promising. Further, expression of the channels cloned from the zebrafish in heterologous systems [15,41,42] provides an attractive platform for electrophysiological studies since dissociated hair cells from the inner ear of the zebrafish are extremely difficult to patch (Moore, unpublished observations, 2010; however, see [43]).

## Conclusions

Recent advances in sequencing the zebrafish genome have provided further insight into modeling human diseases [44,45]. Nevertheless, the chromosomal localizations and/or complete sequencing of the *kcnq4* gene remain to be completed. Western blots demonstrated that the Kcnq4 protein is expressed in the brain as well as the ear. Thus, using the zebrafish with its rapid developmental period as a laboratory specimen may accelerate genetic screening for more specific KCNQ channel mutants, and perhaps foster drug discovery strategies for chemotherapeutic intervention in diseases associated with mutations in the Kv (x) family of genes, e.g., conditions manifested in humans such as hearing loss, and tinnitus.

## Methods

### Animals

Animal procedures were approved by the NU-ACUC (Approval number 2006 - 1034) and were performed in accordance with regulations for the care and use of laboratory animals. Adult zebrafish (initial stock was a kind gift from Dr. Jacek Topczewski, Ann Lurie Children’s Research Medical Center, Northwestern University, Chicago, IL) were kept in an aquarium that was maintained at 25°C, filtrated, pH balanced, with frequent removal of excess nitrate, nitrite, ammonia, chloramines, and chloride. Exchange of conditioned tap water occurred at regular intervals. Two bottom feeder fish (Bristle nose catfish, *Ancistrus temmincki*) were kept in the aquarium to reduce the accumulation of waste. Animals were fed twice daily using a combination of flake or morsels that had been sterilized (UV illumination overnight) before usage. Wild type embryos were collected from natural matings in our lab, or ordered from ZIRC (University of Oregon, Eugene, OR), and were kept in 12-well clusters (~n = 6 each well) at 28.5°C in an air-only incubator. Stages were referred to in hours post-fertilization (hpf) or days post-fertilization (dpf) [9]. After 24-hpf, some larvae were maintained in 0.03% phenylthiourea to prevent melanin pigment formation [46] to ease the identification of a normally developed lateral line.

### Tissue extraction

Zebrafish were sacrificed using a combination of Tricaine Methylsulphonate (MS-222) and ice, and the various tissues were rapidly removed. Zebrafish brain, heart, and ear were dissected, used immediately for experimentation, or pooled separately in 1.5 ml Cryovials and placed in liquid nitrogen until use. Total RNA was extracted from the tissues using TRIzol reagent (Invitrogen, Carlsbad, CA). Ear tissues were placed in 6.0 ml of low calcium saline (LCS, 10 mM HEPES, 100 μM CaCl2, 110 mM NaCl, 2.0 mM KCl, 2.0 mM MgCl2, 3.0 mM D-glucose, pH 7.3). EDTA and MgCl_2_ (12 μl) were added to the ear tissues and incubated for 15 min to prevent calcium carbonate leakage from the inner ear otolithic structures.

### RT-PCR and PCR

Primers for *kcnq1-5* and controls were designed based on zebrafish DNA sequences found in publically available databases such as the NCBI (GenBank), and Ensembl. The nucleotide sequence was searched using BLASTN to determine the number and location of different exons. Primers were designed using PrimerQuest (IDT, Coralville, IA). Nested primers were designed to cross exon boundaries and selected to amplify a 200 – 600 bp fragment of the desired *kcnq* mRNA. Primers were selected for optimum base content and annealing properties to the desired mRNA. Searching the zebrafish genome and expressed mRNAs was conducted using the BLAST suite of programs [47].

Total RNA pellets were washed with 75% ethanol, dried, suspended in RNAse free water and stored at a temperature of 4°C. The RNA concentration and purity was quantified by spectrophotometry (Beckman DU-7500, Fullerton, CA) using the absorbance ratio of A260/A280. Individual PCR reactions contained forward and reverse primers for the desired target, and control reactions contained primers for β-actin. The one-step RT-PCR system (Invitrogen, Carlsbad, CA) was used for amplification of target mRNAs. For each reaction, 200 - 400 ng of total RNA was used for the RT-PCR.

Reactions were performed in a thermocycler for 30 cycles (Techne, TC-312, Minneapolis, MN) with recommended denaturation (94°C, 2 min), annealing (55°C, 30 s), extension (72°C, 2 min), and hold (72°C, forever). The PCR products were separated using 1.0 – 2.0% agarose gel electrophoresis with gels containing ethidium bromide. Gel photographs were taken (Kodak, DC 290, Rochester, NY), transferred and stored to a microcomputer (Dell Dimension 8200). The molecular ladder (M) of the gels was separated by 100 bp bands with the first band at the bottom being 100 bp; the brightest band near the top of the ladder is at 600 bp.

### Sequencing of kcnq4 PCR products

Gel bands were excised and purified using purification columns (Catalog #K2100, Invitrogen, Carlsbad, CA) designed for agarose gel extracts. The purified DNA products were sequenced at Sequetech (Mountain View, CA). The 5′ PCR primers for each product were used as sequencing primers (see Table 1).

### Quantitative RTPCR (qRTPCR)

qRTPCR was conducted using a Biorad MyIQ detection system. Second strand synthesis was conducted using the IScript cDNA synthesis kit (Biorad, Grand Island, NY) starting with 0.45 to 0.74 μg of mRNA from the target tissues (brain, ear, or heart). An aliquot of the cDNA (5-10 ng) was then subjected to quantitative PCR in 96 well plates in the BioRAD Icycler using IQ supermix (Biorad, Grand Island, NY) and primers (100 nM each) selected to generate 100-200 bp amplicons for *kcnq1-5*. GAPDH was used as an internal control (Table 4). After initial denaturation, samples were subjected to 40 cycles of PCR (95°: 15 s, 60°: 60 s) with SYBR green dye fluorescence read at the end of each cycle. Melting curves were obtained at the end of the runs to verify that single melting point species were generated in each reaction. Well factors were collected to compensate for differences in responses across the plate. Cycle times were calculated, and data were transferred to a spreadsheet for calculation of deltaCTs. Dilutions of the mRNA (1:2, 1:10, 1:50, and 1:250) of the mixed cDNA were used to determine amplification efficiency, as reported previously [48].

**Table 4:** Sequences of primers used for qRTPCR

**Primer ID**	**Sequence 5′ – 3′**	**Sequence source**
KCNQ1 FOR	TTC ACA GGG CCA TCT CAA CCT CAT	NM_001123242
KCNQ1 REV	TCA AGC GCT CTG AAC TTG TCT GGA	
KCNQ2 FOR	TCA GCG GAT TCA GCA TCT CAC AGT	XM_003198845
KCNQ2 REV	TGT CCG ATT CAC CCT CTG CAA TGT	
KCNQ3 FOR	AAC TCC ATT TCC GTA CCC ATC CCA	XM003197933
KCNQ3 REV	TGT CTC TCC ACC CGC ACA AAT CTA	
KCNQ4 FOR	TTT CGC ACA TCT CTG CGC CTC AAA	ENSDART00000125606
KCNQ4 REV	TCC ATG GCC ACG TCA CAG TAA GAT	
KCNQ5aFOR	ACA ACC AAC CTT CCA GTC CAG ACA	XM_679763.4
KCNQ5a REV	TAA CTC ACT AAA CCG CTG GTG GCT	
GAPDH FOR	TTG CCG TTC ATC CAT CTT TGA CGC	NM_001115114
GAPDH REV	TCA GGT CAC ATA CAC GGT TGC TGT	

### Antibody preparation

A synthetic peptide CSGKMGFRDRIRMNNSRSS based upon the reported partial cDNA, and putative amino acid sequences for zebrafish *Kcnq4* was prepared and conjugated to Kehoe Limpet hemocyanin (KLH) for immunization. Two rabbits were used for antibody production by Bio-Synthesis (Lewisberg, TX). Antisera were obtained 6 -10 weeks after immunization and characterized for immunoreactivity against zebrafish tissue extracts.

### Western blotting

Zebrafish brain and ear were homogenized in a lysis buffer (20 mM Tris, 150 mM NaCl, 1.0 mM EDTA, 1.0 mM EGTA, 2.5 mM NaPyrophosphate, 1.0 mM Na Vanadate, 1.0 mM betaglycerol phosphate, 1.0 mg/mL leupeptin, and 1.0% Triton X-100, pH 7.5) using a motor driven pestle (5 bursts of 10 s) on ice. The crude homogenate was centrifuged at 1.4 × 10^3^ rpm (Eppendorf microfuge, Hauppauge, NY). The supernatant fraction was removed, saved and the pellet extracted again using lysis buffer plus 1.0% SDS. After centrifugation to remove insoluble material, the soluble pellet fraction was saved. Protein concentrations in each fraction were determined using a reagent (Pierce BCA, Pittsburgh, PA) as suggested by the manufacturer. Electrophoresis was conducted on 4 - 12% SDS Page minigels (Invitrogen, Grand Island, NY). A total of 25 - 30 μg of brain fractions, ear and heart fractions (10 - 12 μg) were loaded into separate lanes on the gel. After electrophoresis, the gel was blotted to PVDF membranes (Millipore, Billerica, MA) using a Biorad transfer cell and Tris-Glycine-20% methanol transfer buffer. Transfer was accomplished at a constant voltage (40 V) for 1.5 hrs at room temperature. Membranes were blocked in 5.0% nonfat dry milk in Tris-buffered saline (20 mM Tris -150 mM NaCl, pH 7.5) containing 0.1% Tween 20 (TBS-T). Blots were then treated with anti-Kcnq4 peptide antiserum (1:2000) in TBS-T with 5.0% nonfat dry milk at room temperature for 2.0 hrs. After washing 5x with TBS-T, the blots were then incubated with HRP-conjugated Goat anti-rabbit antibody (Bio-Rad, 1:5000) for 1.0 hr at room temperature. After washing 5x with TBS-T, the blot was developed with electrochemiluminescent substrate (Pierce West Pico, Pittsburgh, PA) and bands were detected on film (Kodak Biomax, Rochester, NY).

### Data analysis

Expression intensity of gene transcripts was analyzed using Image J (NIH Image, Bethesda, MD). Data display was accomplished using Origin (v8.0 Origin Software, Northampton, MA).

## Competing interests

The authors declare no financial or non-financial competing interests.

## Authors’ contributions

Conceived idea for research (EJM, TJL), designed research (EJM, TJL, CW), performed experiments (CW, KS, KL, MH, CT), data analysis (CW, TJL, EJM), wrote the paper (EJM, TJL, CW). All authors read and approved the final manuscript.
